# Retraction of: A fungal-derived inducer amplifies GABA biosynthesis to protect tea from heat

**DOI:** 10.1093/plphys/kiag360

**Published:** 2026-08-04

This is a retraction of Bo Xu, A fungal-derived inducer amplifies GABA biosynthesis to protect tea from heat, *Plant Physiology*, 2026, kiag231, https://doi.org/10.1093/plphys/kiag231.

This News & Views piece is being retracted by the journal's Editor-in-Chief because it comments in detail on a research article that was submitted to but ultimately not published in this journal. This retraction in no way suggests any misconduct or fault on the part of the author. The journal sincerely apologizes to the author of this piece and to its readers for any inconvenience that the publication and subsequent retraction of this paper may have caused.

